# Impact of the Four-Hour Rule in Western Australian hospitals: Trend analysis of a large record linkage study 2002-2013

**DOI:** 10.1371/journal.pone.0193902

**Published:** 2018-03-14

**Authors:** Hanh Ngo, Roberto Forero, David Mountain, Daniel Fatovich, Wing Nicola Man, Peter Sprivulis, Mohammed Mohsin, Sam Toloo, Antonio Celenza, Gerard Fitzgerald, Sally McCarthy, Ken Hillman, on behalf of the Four-Hour Rule Partnership Grant

**Affiliations:** 1 Emergency Medicine, University of Western Australia, Nedlands, Perth, WA, Australia; 2 Simpson Centre for Health Services Research, UNSW Australia, SWS Clinical School, Liverpool, Sydney, NSW, Australia; 3 Ingham Institute for Applied Research. Liverpool Hospital, Liverpool, Sydney, NSW, Australia; 4 Sir Charles Gairdner Hospital, Nedlands, Perth, WA, Australia; 5 Australasian College for Emergency Medicine, West Melbourne, Melbourne, VIC, Australia; 6 Royal Perth Hospital, Perth, WA, Australia; 7 Centre for Clinical Research in Emergency, University of Western Australia, Nedlands, Perth, WA, Australia; 8 Psychiatry Research and Teaching Unit, SWSLHD, NSW Health, Liverpool, Sydney, NSW, Australia; 9 School of Psychiatry, Faculty of Medicine, UNSW, Kensington, Sydney, NSW, Australia; 10 School of Public Health and Social Work, Queensland University of Technology, Kelvin Grove, Brisbane, QLD, Australia; 11 Emergency Care Institute, NSW Agency for Clinical Innovation, Chatswood, Sydney, NSW, Australia; 12 Prince of Wales Clinical School, UNSW, Randwick, Sydney, NSW Australia; Public Library of Science, UNITED KINGDOM

## Abstract

**Background:**

In 2009, the Western Australian (WA) Government introduced the Four-Hour Rule (FHR) program. The policy stated that most patients presenting to Emergency Departments (EDs) were to be seen and either admitted, transferred, or discharged within 4 hours. This study utilised de-identified data from five participating hospitals, before and after FHR implementation, to assess the impact of the FHR on several areas of ED functioning.

**Methods:**

A state (WA) population-based intervention study design, using longitudinal data obtained from administrative health databases via record linkage methodology, and interrupted time series analysis technique.

**Findings:**

There were 3,214,802 ED presentations, corresponding to 1,203,513 ED patients.

After the FHR implementation, access block for patients admitted through ED for all five sites showed a significant reduction of up to 13.2% (Rate Ratio 0.868, 95%CI 0.814, 0.925) per quarter. Rate of ED attendances for most hospitals continued to rise throughout the entire study period and were unaffected by the FHR, except for one hospital. Pattern of change in ED re-attendance rate post-FHR was similar to pre-FHR, but the trend reduced for two hospitals. ED occupancy was reduced by 6.2% per quarter post-FHR for the most ‘crowded’ ED. ED length of stay and ED efficiency improved in four hospitals and deteriorated in one hospital. Time to being seen by ED clinician and Did-Not-Wait rate improved for some hospitals. Admission rates in post-FHR increased, by up to 1% per quarter, for two hospitals where the pre-FHR trend was decreasing.

**Conclusions:**

The FHR had a consistent effect on ‘flow’ measures: significantly reducing ED overcrowding and access block and enhancing ED efficiency. Time-based outcome measures mostly improved with the FHR. There is some evidence of increased ED attendance, but no evidence of increased ED re-attendance. Effects on patient disposition status were mixed. Overall, this reflects the value of investing resources into the ED/hospital system to improve efficiency and patient experience. Further research is required to illuminate the exact mechanisms of the effects of FHR on the ED and hospital functioning across Australia.

## Introduction

Access Block and Emergency Department (ED) overcrowding remain a major problem for patients, professionals and public health care systems worldwide. Adverse effects of these phenomena are numerous, wide-ranging and well described in the literature[1–5]. These include increased mortality and morbidity, delayed pain relief and critical interventions, longer hospital stays, increased aggression in EDs, delayed ambulance offloads with poorer ambulance response times, and overall poor experience for patients, carers and staff in EDs. These have been associated with negative reports from hospitals and governments alike [6–9].

In 2000, responding to these pressures, the United Kingdom (UK) Blair Government developed a health plan containing the first version of the “Four-Hour Rule” (FHR) where 100% of patients were expected to be admitted, transferred or discharged from the ED within four hours of arrival at ED [10, 11]. In 2005, the UK National Health Service (NHS) reported significant improvements and that they had overall achieved a modified 98% target [12]. Putative reasons for the achievement, backed by minimal research, included better management, more resources, changed work flows, faster admissions to wards, better discharge processes and a stronger (enforced) managerial commitment to removing bottlenecks [7, 8, 12–14]. However, reports also indicated the FHR had put significant numbers of patients at serious risk of harm [15, 16]. Partly as a result of these problems, the UK FHR was modified with a reduced 95% FHR target plus additional quality measures [13, 17, 18].

Learning from the UK experience, the state of Western Australia (WA) introduced a modified version of the FHR program, using gradual incremental targets and multiple quality measures, to its four tertiary hospitals (one paediatric, two adult, and one mixed) with EDs on 14 April 2009. The original target of this Stage 1 required that by April 2011, 98% of patients presenting to ED would be seen and be discharged, admitted, or transferred within 4 hours of their arrival[19, 20]. Due to several challenges, the target was revised to 85% for two adult, one mixed (adult and paediatric) hospitals, but kept at 98% for the paediatric hospital[19]. Stage 2 of the policy involved the state’s general hospitals in the metropolitan area, introducing the FHR from 26 October 2009. From 2011 onwards, a FHR was adopted across Australia, known as the National Emergency Access Target (NEAT), which required a target of 90% of ED presentations to be admitted, discharged or transferred within 4 hours of arrival to be reached by 2015 [21].

This study assessed the impact of the WA FHR program on multiple measures of ED functioning and patient outcomes for five de-identified participating hospitals, including all four tertiary hospitals from Stage 1 and one general hospital from Stage 2.

## Materials and methods

### Design

This was a population-based intervention study, using de-identified record-linkage health data in Western Australia between 2002 and 2013.

### Participants and data source

The study utilised the WA Emergency Department Data Collection (EDDC) and the WA Hospital Morbidity Data Collection (HMDC), of which WA Department of Health is the custodian. The EDDC contained all 3,219,905 ED presentations, corresponding to 1,206,466 patients, to the five-participating hospital EDs between 1 January 2002 and 31 December 2013, except for Hospital E whose data were included from 1 July 2004 (when this hospital moved to the same ED information system as the other four hospitals). The HMDC was used for identifying hospital admissions following ED presentations (see *Data definitions* below).

### Outcome measures

Ten outcome measures (five principal and five secondary) are assessed in this study. The five principal measures are: Access Block (defined as ED length of stay (EDLOS) longer than 8 hours) for an admitted patient, ED occupancy rate, ED attendances and re-attendance (within 7 days of index ED discharge) and ED length of stay (EDLOS). These are designated ‘principal’ as they are considered as the direct markers of the intended outcomes or changes expected of the FHR. Secondary measures include: time to be seen by ED clinician, ED efficiency (indicated by the percentage of ED attendances with EDLOS ≤ 4 hours), Did-Not-Wait (DNW–left ED before being seen by an ED clinician), admission, and short-stay admission (i.e., with discharge within 24 hours).

### Data definitions

Identification of a hospital admission subsequent to an ED attendance was via match-merging records from the EDDC and HMDC. Records from the HMDC would need to have their status flagged as ‘emergency’ admissions to be considered in the match-merging process with EDDC records. The timeframe that allowed for an ED-Morbidity match was ED discharge time being within [-2 hours, +1 hour] of the subsequent hospital admission time. The absolute value of 1-hour buffer time was included to take into account the potential inaccuracy/imprecision of the time recorded in the HMDC; as the data custodian recommended these times be rounded up to the hour unit. One extra hour was allowed after ED discharge time and before hospital admission time, taking into account logistic reasons, such as time taken for finalizing hospital paperwork and/or transfer to another hospital for admission.

ED occupancy rate served as an index of ED overcrowding, whereby ED occupancy rate greater than 100% indicates an overcrowded ED environment. It was calculated for every one-hour block throughout the study period for each hospital ED, and defined as the total ED patient count divided by the concerned ED’s functional capacity. Functional capacity are numerical values agreed by the Western Australian Department of Health, the ED concerned and the respective hospital, as the volume of patients that the ED can functionally receive and treat at a given point in time.

### Data analysis

Data were first summarised in quarters and presented graphically as time series for the 10 outcomes separately. The impact of the FHR on these ED outcome measures was assessed statistically using Interrupted Time Series (ITS) analysis, which is the recommended technique for analysing data from a population-based interventional study such as this one [22–24]. Several assumptions of the data need to hold for the ITS analysis to be applied correctly [25]. Among these, the following four key assumptions were checked and satisfied for our data. First, the outcome data (i.e., dependent variable) are of continuous or discrete (e.g., count) type. Second, the relationship between the outcome/dependent variable and the explanatory/independent variables (usually related to the intervention and its timeframe) is linear, or can be transformed to be so. Third, ideally there needs to be at least 12 data (time) points before intervention and 12 points after intervention, to ensure a reasonable degree of accuracy and reliability in trend detection and estimation. Fourth, within each data point, a minimum of 100 observations is required, to ensure “an acceptable level of variability of the estimate” at each point [25].

In the ITS analysis, the pre-FHR period was comprised of 29 Quarters (14 January 2002 to 13 April 2009 inclusive) for the four Stage 1 hospitals, and 21 Quarters (26 July 2004 to 25 October 2009 inclusive) for the Stage 2 hospital. Similarly, the post-FHR period spanned over 18 Quarters (14 April 2009 to 13 October 2013 inclusive) for the four Stage 1 hospitals, and 16 Quarters (26 October 2009–25 October 2013) for the Stage 2 hospital. Results from these analyses are reported in relation to: (i) trend pre-intervention, (ii) immediate change in levels from pre-intervention to post-intervention, and (iii) change in trend post-intervention.

Data on Access Block were log-transformed before being subjected to the ITS analysis, and then back-transformed (i.e., exponentiated) once modelled, to ensure the predicted values derived from the statistical models were non-negative. This consideration was not required for the ITS analysis of the other nine outcomes, where data in (original) normal base were used. Accordingly, estimates derived from the ITS models are to be interpreted as “differences” for these nine outcomes, and as “ratios” for Access Block.

Inferential statistics presented in this paper are accompanied by the respective 95% Confidence Intervals (CI). Statistical significance levels are not adjusted for multiple outcome analyses in this study, because such adjustments are argued by some as unnecessary and potentially misleading [26, 27]. However, where effects are found to be of strong statistical significance (p<0.001), these are also denoted accordingly in the presentation of the results.

Data were managed and analysed using SAS software version 9.3.

### Ethics clearance

The study received ethics approval from the respective Human Research Ethics Committees of Western Australian Department of Health, University of New South Wales, and The University of Western Australia, as well as governance approval from all participating hospitals.

## Results

### Patient (and system) characteristics

Checks for data completeness and validity led to the removal of 5,103 ineligible records (0.16% of all available records). Of these, 4,568 (89.5%) were coded as ‘Dead on Arrival’, while the remaining 535 records had missing, duplicated, or invalid time measures (e.g., discharge time recorded as before presentation time). Accordingly, a total of 3,214,802 ED presentation records, corresponding to 1,203,513 persons, were deemed eligible and retained for reporting in this study.

Table 1 describes the socio-demographic, clinical and environmental characteristics of all eligible ED attendances during the study period 2002–2013. Gender distribution was similar across all five hospitals, with a slightly higher percentage of males. Over 90% of the ED attendances were by residents of the Perth metropolitan area, where the participating hospitals were located. Distribution of socio-economic disadvantage varied somewhat with the hospital locations. The clinical profile was mostly similar for all hospitals, with up to one-third of the presentations being due to injuries, and approximately one-third of the presentations using ambulance transport. Triage categories were distributed similarly for most hospitals, with Hospitals C and E appearing to have lower proportions of high-acuity cases. Proportions of attendances during ‘Day shift’ in general were higher than ‘Evening’ or ‘Night shifts’. Distributions of attendances across the other three time-related factors (influenza season, weekend, and holiday) were similar for the five hospitals.

**Table 1:** Socio-demographic, clinical and environmental characteristics of ED patients and presentations by hospital.

	Number (%)				
Characteristics	Hospital A	Hospital B	Hospital C	Hospital D	Hospital E
	(N = 738714)	(N = 573577)	(N = 664415)	(N = 613864)	(N = 624232)
**Demographic** ^a^					
Gender (Male)	417715 (56.6)	309293 (53.9)	370589 (55.8)	310361 (50.6)	316074 (50.6)
Remoteness					
Perth metropolitan	555111 (91.0)	471008 (92.5)	593811 (93.4)	537009 (94.7)	542040 (93.6)
Inner Regional	23007 (3.8)	30019 (5.9)	24538 (3.9)	15117 (2.7)	29862 (5.2)
Outer Regional, Remote, Very Remote	31828 (5.2)	8218 (1.6)	17505 (2.8)	15189 (2.7)	7224 (1.3)
Socio-economic disadvantage (Yes)	277887 (41.4)	226288 (41.9)	220751 (33.8)	143275 (23.8)	73120 (12.3)
**Clinical**					
Arrived at ED by Ambulance (Yes)	261538 (35.4)	171658 (29.9)	43283 (6.5)	230869 (37.6)	87074 (14.0)
Triage category					
1: Immediate	15857 (2.2)	5391 (0.9)	2509 (0.4)	9888 (1.6)	3380 (0.5)
2: Emergency	135424 (18.3)	71650 (12.5)	19635 (3.0)	123982 (20.2)	69147 (11.1)
3: Urgent	246027 (33.3)	206861 (36.1)	142414 (21.4)	263289 (42.9)	191296 (30.7)
4: Semi-urgent	297987 (40.3)	236283 (41.2)	483739 (72.8)	198005 (32.3)	337370 (54.1)
5: Non-urgent	43235 (5.9)	53374 (9.3)	16050 (2.4)	18700 (3.1)	23039 (3.7)
ED diagnosis grouping ^b^					
Cardiovascular	55206 (7.6)	32810 (6.1)	3532 (0.5)	48059 (7.8)	23669 (3.9)
Respiratory	39226 (5.4)	37739 (7.0)	117057 (17.7)	35612 (5.8)	49600 (8.2)
Neurological	15336 (2.1)	9898 (1.8)	7902 (1.2)	16180 (2.6)	9740 (1.6)
Psychiatric	35861 (4.9)	20328 (3.8)	9565 (1.5)	38613 (6.3)	18553 (3.1)
Injury	222076 (30.5)	172186 (31.8)	170450 (25.8)	162072 (26.5)	210842 (35.0)
Miscellaneous	57255 (7.9)	39750 (7.3)	26403 (4.0)	49363 (8.1)	48073 (8.0)
Other	303074 (41.6)	229564 (42.3)	324840 (49.2)	262815 (42.9)	241291 (40.1)
**Environmental**					
ED shift					
Day (08:00–15:59)	314651 (42.6)	254967 (44.5)	284724 (42.9)	280268 (45.7)	278500 (44.6)
Evening (16:00–23:59)	285101 (38.6)	224604 (39.2)	299836 (45.1)	236029 (38.5)	248626 (39.8)
Night (00:00–07:59)	138962 (18.8)	94006 (16.4)	79855 (12.0)	97567 (15.9)	97106 (15.6)
Influenza & RSV season (Yes) ^c^	323524 (43.8)	251488 (43.9)	331319 (49.9)	276055 (45.0)	312482 (50.1)
Weekend (Yes) ^d^	270762 (36.7)	212936 (37.1)	249020 (37.5)	218407 (35.6)	228985 (36.7)
Holiday (Yes) ^e^	82316 (11.1)	64287 (11.2)	59602 (9.0)	67612 (11.0)	66144 (10.6)

^a^ Information on patients' age is not reported due to restrictions on data release and project funding.

^b^ Diagnostic grouping as per ICD-10 classifications. Miscellaneous common conditions include shortness of breath, chest pain, and stroke.

^c^ RSV: respiratory syncytial virus.

^d^ Weekend starts from 18:00 Friday and ends at 08:00 Monday.

^e^ Holiday season starts from 24 December and ends 31 January

**Fig 1:**
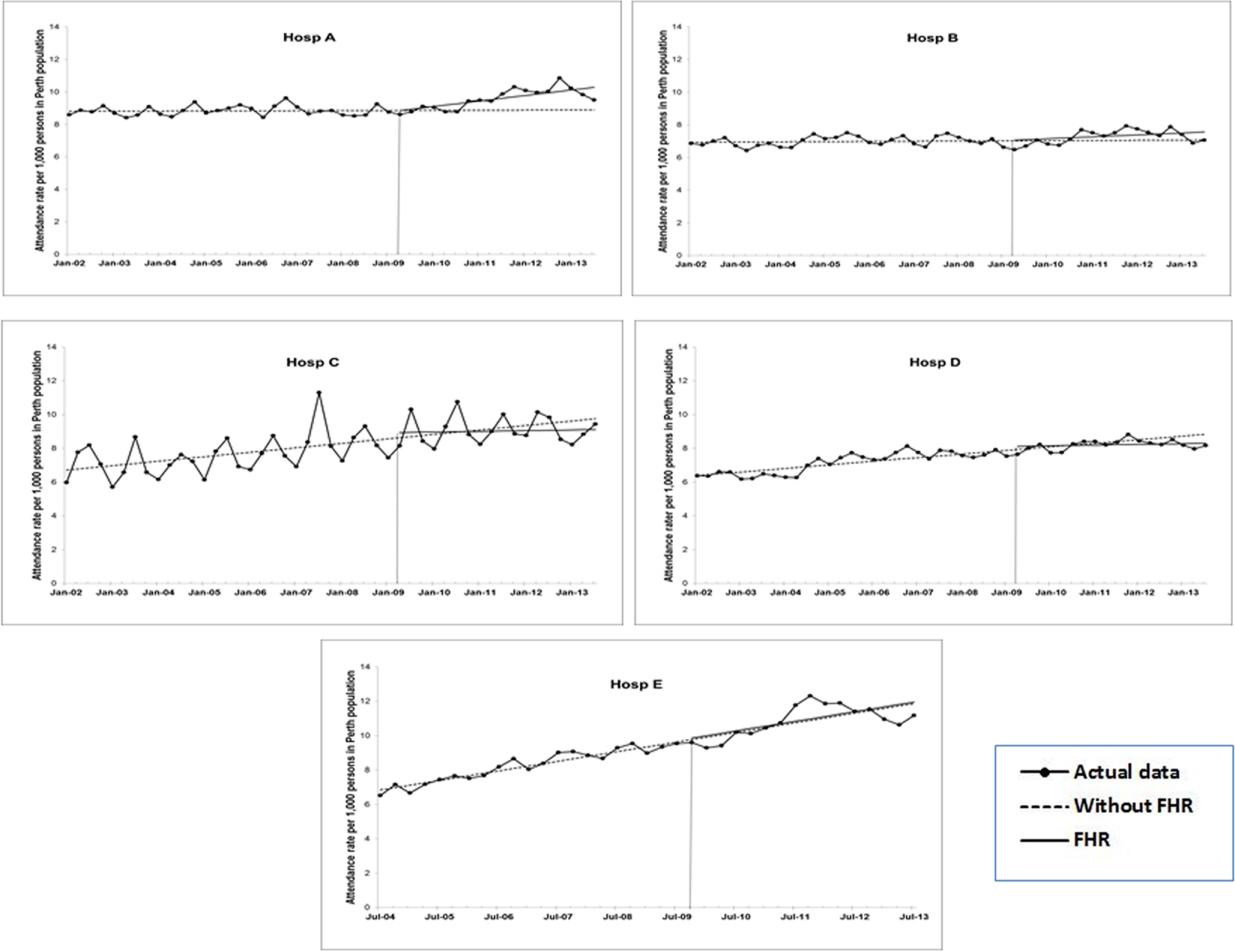
ITS analysis for rate of ED attendance across WA hospitals (A to E). Estimated trend lines for Without FHR (broken line) and With FHR (solid line). Series with markers represent actual data.

**Fig 2:**
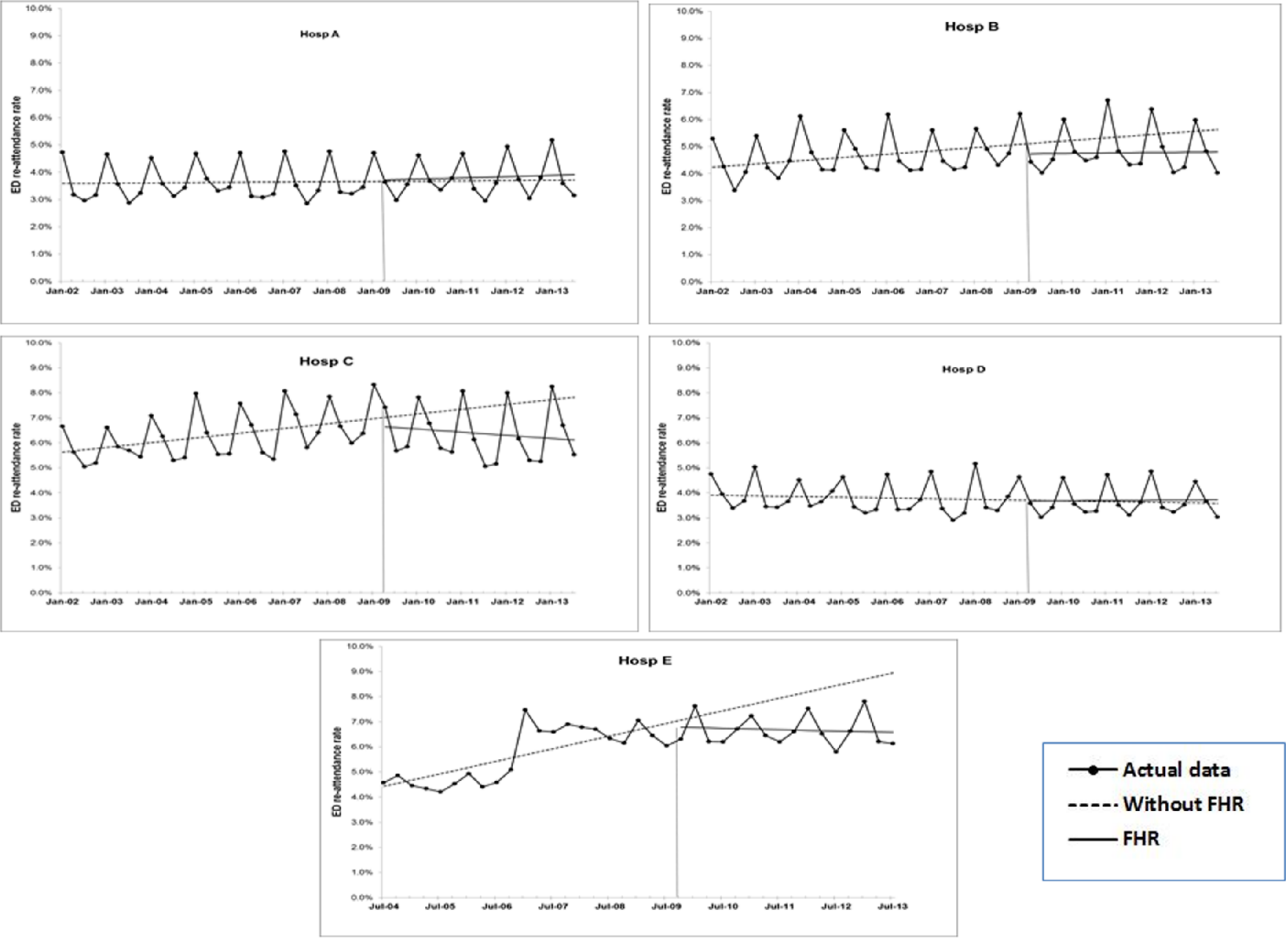
Results of ITS analysis for rate of ED re-attendance across hospitals: Estimated trend lines for Without FHR (broken line) and with FHR (solid line). Series with markers represent actual data.

**Fig 3:**
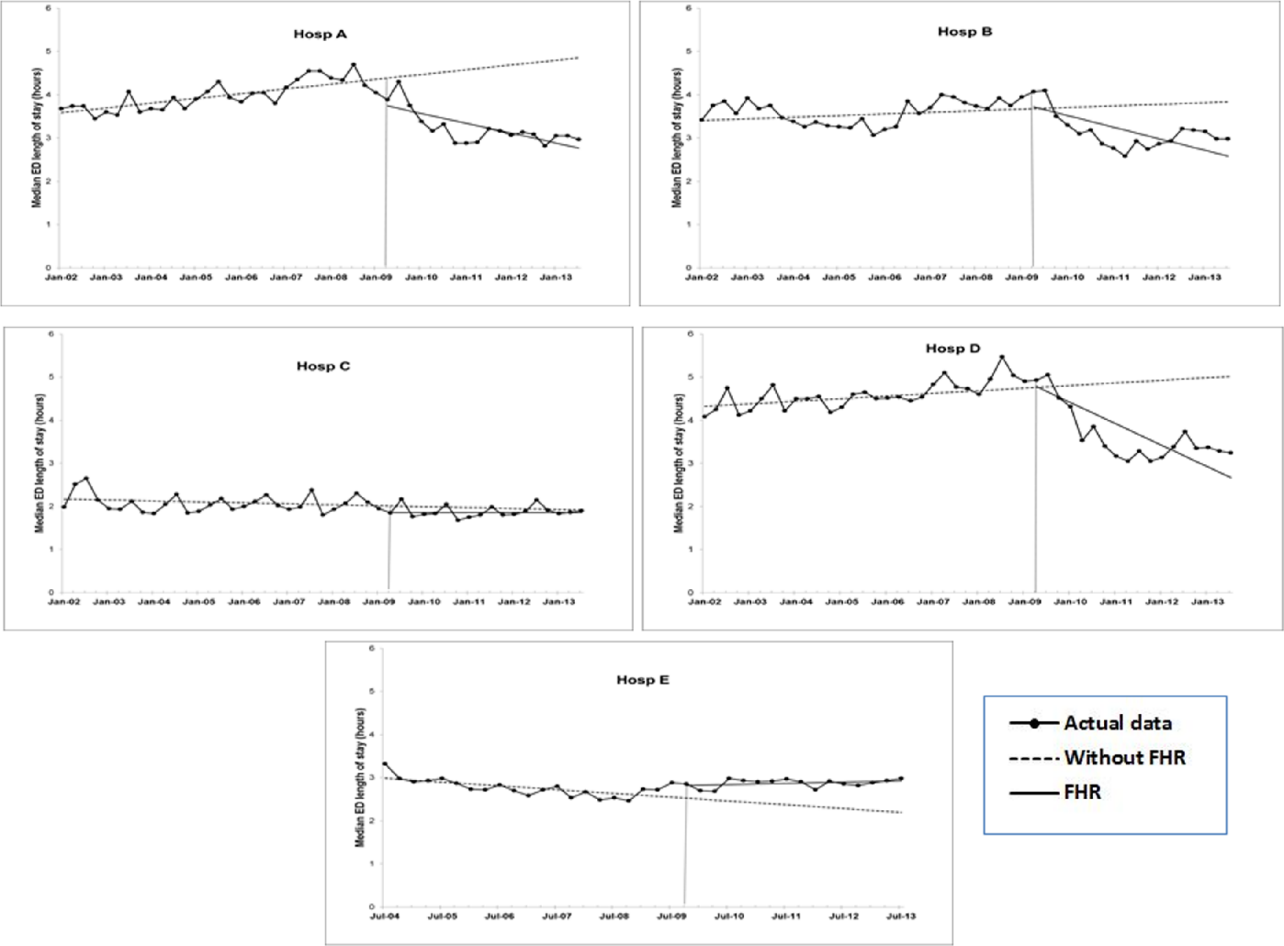
ITS analysis for median length of stay in ED (measured in hours): Estimated trend lines for Without FHR (broken line) and with FHR (solid line). Series with markers represent actual data.

**Fig 4:**
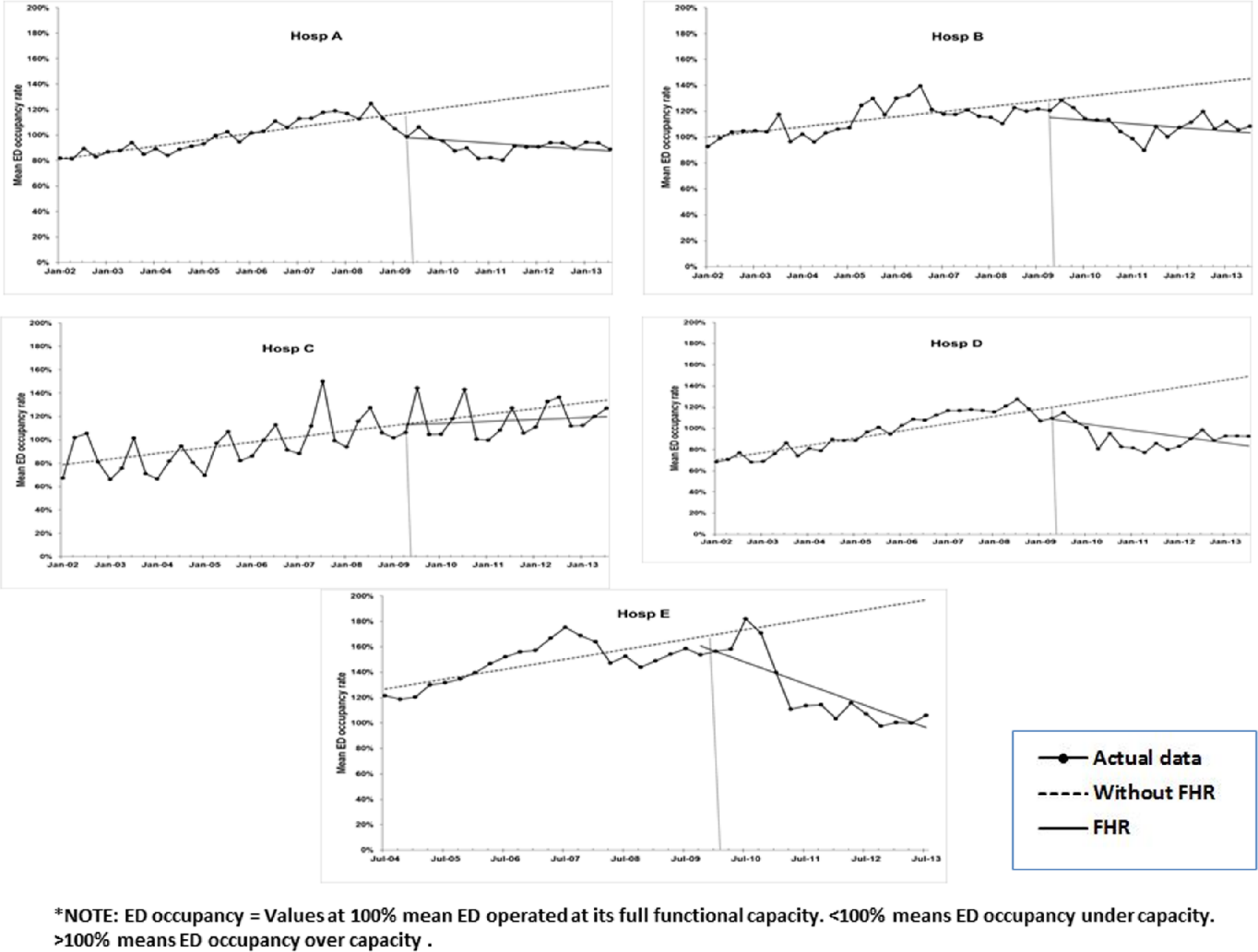
Results of ITS analysis for mean ED occupancy rate: Estimated trend lines for Without FHR (broken line) and with FHR (solid line). Series with markers represent actual data.

**Fig 5:**
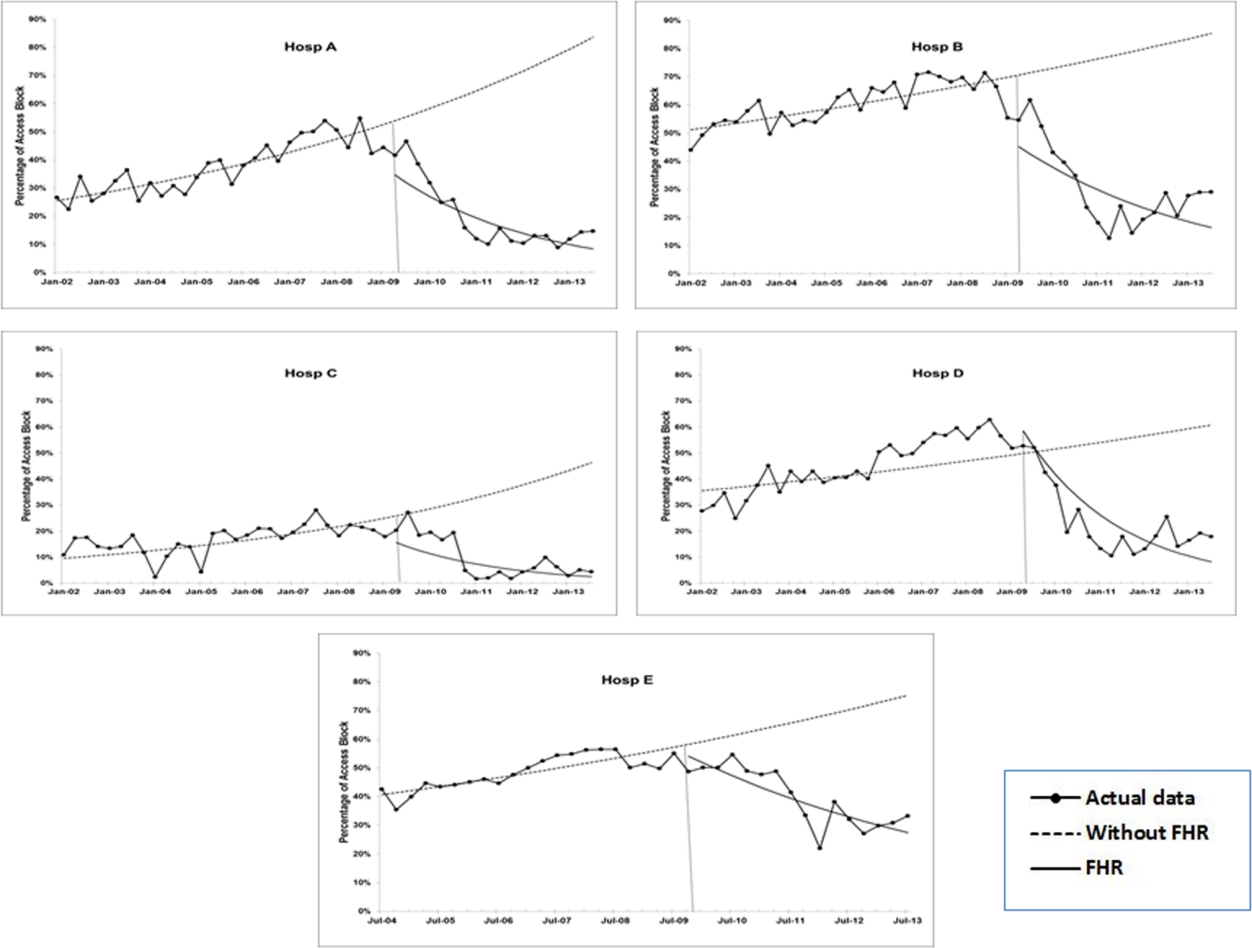
Results of ITS analysis for rate of access block: Estimated trend lines for Without FHR (broken line) and with FHR (solid line). Series with markers represent actual data.

Table 2 provides a summary of results from the ITS analyses. These are further described below, in conjunction with Figs 1–5. Supporting information and a brief description of the ITS technique, modelling strategy and interpretation of results are presented below [26NR, 27] **(S1 File)**.

**Table 2:** Summary results per hospital of attendance rates, time in ED, flow and disposition.

			Estimate (95% CI)		
Measure Type	Outcome	Hospital	Pre-FHR Trend ^a^	FHR Change in Level ^b^	FHR Change in Trend ^c^
**Attendance**	Rate of	A	0.18% (-1.56%,1.91%)	-8.78% (-55.66%,38.11%)	8.27% (3.91%,12.62%)**
	Attendance	B	0.29% (-1.16%,1.74%)	1.73% (-33.22%,36.68%)	2.62% (-1.02%,6.26%)
		C	6.62% (4.05%,9.18%)** ^d^	36.03% (-32.08%,104.14%)	-5.57% (-11.50%,0.37%)
		D	5.32% (2.82%,7.82%)**	23.31% (-21.92%,68.54%)	-4.28% (-9.73%,1.18%)
		E	13.97% (10.11%,17.83%)**	9.34% (-64.10%,82.77%)	-0.01% (-8.12%,8.09%)
	Percentage of	A	0.00% (0.00%,0.01%)	0.04% (-0.17%,0.25%)	0.01% (-0.01%,0.03%)
	Re-Attendance	B	0.03% (0.00%,0.06%)* ^d^	-0.35% (-0.71%,0.01%)	-0.03% (-0.08%,0.03%)
	in 7 days	C	0.05% (0.04%,0.06%)**	-0.29% (-0.62%,0.04%)	-0.08% (-0.11%,-0.05%)**
		D	-0.01% (-0.01%,0.00%)	-0.03% (-0.24%,0.17%)	0.01% (-0.01%,0.03%)
		E	0.13% (0.06%,0.19%)**	-0.14% (-1.21%,0.93%)	-0.14% (-0.26%,-0.02%)*
**Time**	Median	A	0.028 (0.012,0.043)**	-0.559 (-0.946,-0.171)*	-0.085 (-0.119,-0.051)**
	EDLOS	B	0.009 (-0.008,0.027)	0.118 (-0.281,0.517)	-0.076 (-0.123,-0.030)*
	(hours)	C	-0.006 (-0.018,0.007)	-0.157 (-0.399,0.084)	0.005 (-0.024,0.034)
		D	0.015 (-0.009,0.039)	0.159 (-0.302,0.620)	-0.139 (-0.205,-0.073)**
		E	-0.022 (-0.031,-0.013)*	0.266 (0.094,0.439)*	0.029 (0.012,0.045)*
	Median Time	A	0.634 (0.451,0.817)**	-15.519 (-20.550,-10.489)**	-0.540 (-0.956,-0.123)*
	to Be Seen	B	-0.226 (-0.715,0.264)	-4.049 (-14.432,6.334)	0.327 (-0.789,1.443)
	(minutes)	C	-0.226 (-0.303,-0.149)**	-1.511 (-3.498,0.476)	0.536 (0.362,0.709)**
		D	0.574 (0.403,0.746)**	-0.867 (-5.073,3.340)	-1.031 (-1.422,-0.640)**
		E	0.288 (0.125,0.450)*	-0.870 (-4.192,2.451)	0.169 (-0.117,0.455)
**Flow**	Mean	A	1.25% (0.84%,1.66%)**	-17.58% (-27.26%,-7.90%)**	-1.88% (-2.81%,-0.95%)**
	ED Occupancy	B	0.98% (0.54%,1.42%)**	-11.83% (-24.07%,0.41%)	-1.67% (-2.72%,-0.62%)*
		C	1.20% (0.64%,1.76%)**	0.23% (-12.19%,12.65%)	-0.80% (-2.01%,0.41%)
		D	1.71% (1.00%,2.42%)**	-8.23% (-22.49%,6.03%)	-3.19% (-4.78%,-1.60%)**
		E	1.95% (0.25%,3.65%)*	-0.95% (-22.17%,20.27%)	-6.23% (-9.55%,-2.91%)**
	Percentage of	A	-0.13% (-0.56%,0.29%)	-0.41% (-6.60%,5.79%)	1.86% (0.65%,3.06%)*
	ED Efficiency	B	-0.09% (-0.35%,0.18%)	-3.83% (-8.97%,1.32%)	1.79% (1.03%,2.55%)**
		C	-0.04% (-0.22%,0.15%)	1.70% (-2.12%,5.53%)	0.52% (0.05%,0.98%)*
		D	-0.09% (-0.35%,0.17%)	-3.07% (-9.22%,3.07%)	2.15% (1.38%,2.91%)**
		E	0.18% (0.05%,0.31%)*	-3.40% (-5.80%,-0.99%)*	-0.08% (-0.32%,0.15%)
	Percentage of	A	1.026 (1.016,1.037)**	0.717 (0.526,0.976)*	0.897 (0.873,0.921)**
	Access Block ^e^	B	1.011 (1.000,1.022)*	0.686 (0.494,0.952)*	0.932 (0.904,0.961)**
		C	1.035 (1.008,1.062)*	0.694 (0.324,1.486)	0.868 (0.814,0.925)**
		D	1.012 (0.995,1.029)	1.333 (0.996,1.783)	0.881 (0.836,0.928)**
		E	1.017 (1.008,1.026)**	0.989 (0.840,1.165)	0.939 (0.925,0.954)**
**Disposition**	Percentage of	A	0.05% (-0.08%,0.19%)	2.46% (-0.84%,5.77%)	-0.05% (-0.37%,0.26%)
	Admission	B	0.00% (-0.36%,0.37%)	3.99% (0.32%,7.65%)*	0.01% (-1.23%,1.25%)
		C	-0.18% (-0.24%,-0.13%)**	-0.92% (-2.48%,0.65%)	0.15% (0.02%,0.28%)*
		D	0.07% (-0.03%,0.17%)	6.57% (3.78%,9.36%)**	0.00% (-0.23%,0.23%)
		E	-0.17% (-0.25%,-0.10%)**	-0.41% (-1.86%,1.03%)	0.77% (0.63%,0.90%)**
	Percentage of	A	0.33% (-0.10%,0.76%)	-1.00% (-6.03%,4.04%)	-0.03% (-1.32%,1.27%)
	Short-stay	B	0.20% (-0.35%,0.75%)	2.02% (-3.65%,7.68%)	-0.14% (-1.84%,1.56%)
	Admission	C	0.23% (0.13%,0.32%)**	0.34% (-2.27%,2.94%)	-0.20% (-0.42%,0.01%)
		D	0.27% (0.14%,0.41%)**	8.43% (4.74%,12.13%)**	0.11% (-0.19%,0.42%)
		E	0.20% (0.00%,0.40%)	0.30% (-3.46%,4.07%)	0.38% (0.01%,0.74%)
	Percentage of	A	-0.07% (-0.11%,-0.03%)	-1.21% (-2.08%,-0.35%)	0.03% (-0.05%,0.11%)
	Did Not Wait	B	-0.06% (-0.15%,0.02%)	-1.21% (-3.11%,0.70%)	-0.02% (-0.22%,0.17%)
		C	-0.03% (-0.10%,0.04%)	-0.28% (-1.65%,1.09%)	0.02% (-0.14%,0.17%)
		D	0.00% (-0.03%,0.04%)	-0.42% (-1.33%,0.50%)	-0.11% (-0.19%,-0.03%)*
		E	-0.11% (-0.15%,-0.07%)	-0.12% (-0.96%,0.72%)	0.15% (0.08%,0.23%)**

^a^ Parameter for trend pre-FHR, corresponding to January 2002—April 2009 for (Stage 1) Hospitals A-D, and July 2004—October 2009 for (Stage 2) Hospital E. Estimate is interpreted as magnitude of change per quarter pre-FHR.

^b^ Parameter for change in level, between immediately pre-FHR and immediately post-FHR. Estimate is interpreted as immediate change in the first quarter post-FHR.

^c^ Parameter for change in trend post-FHR, corresponding to April 2009—October 2013 for (Stage 1) Hospitals A-D, and October 2009–2013 for (Stage 2) Hospital E. Estimate is interpreted as magnitude of change in trend per quarter post-FHR, compared to trend pre-FHR.

Est: Estimate. LL: Lower Limit of 95% Confidence Interval. UL: Upper Limit of 95% Confidence Interval.

^d^ (*) and (**) indicate estimates statistically significant at p<0.05 and p<0.001.

^e^ indicates estimates to be interpreted as “ratio”, instead of “difference”.

### Principal outcomes

#### Attendance and re-attendance measures

Rate of ED attendance was on an increasing trend for Hospitals C through E pre-FHR. This increase was as high as 14% (or, on a per 1000 persons basis, this would make 14 per 100,000 population), per quarter for Hospital E (see Table 2). Post-FHR, however, there was no change to the pre-existing trend, except for Hospital A, which observed an increase in attendance rate by 8 per 100,000 population. Attendance rate was most stable for Hospital B, where there was no statistically significant change in the entire study period (Fig 1).

Fig 2, and the relevant statistics in Table 2, indicate a small but persisting and significant increasing trend in rates of ED re-attendance within 7 days for Hospitals B, C, and E, in the pre-FHR period. The FHR did not change the existing trends or levels, except for the reversed (i.e., downward) trend seen for Hospitals C and E. More specifically, taking Hospital E for example, pre-FHR re-attendance rate increased by 0.13% per quarter. However, this trend was reversed by a decrease of 0.14% per quarter post-FHR. There was no statistically significant change in level of re-attendance immediately after the FHR introduction.

#### ED length of stay (EDLOS)

Median EDLOS for Hospital A was on the increase pre-FHR, estimated at a rate of 0.03 hours per quarter (see Table 2). Immediately after the FHR, its EDLOS dropped by approximately 0.6 hour (10 minutes), in the first quarter. The downward trend was initiated and maintained post-FHR (see Hospital A, in Fig 3). The pattern of change in EDLOS at Hospital E (see Hospital E, in Table 2 and Fig 3) was the opposite of that observed at Hospital A. Similar to Hospital A, a significant downward trend in EDLOS was also noted for Hospitals B and D post-FHR. Hospital C displayed no statistically significant changes in EDLOS.

#### ED occupancy and overcrowding

The statistics in Table 2 report a significant pre-FHR increasing trend in ED occupancy rate for all five participating hospitals, which were mostly operating beyond their capacity. The FHR reversed this increasing trend for most hospitals (i.e., except Hospital C). Hospital E demonstrated the largest magnitude of change, with a post-FHR reduction of 6.2% per quarter, overriding its pre-FHR upward trend of 2% per quarter (Fig 4).

#### Access block

Similar to the findings of ED occupancy rate, the FHR completely reversed the pre-existing pattern in access block (Table 2 and Fig 5), which had been increasing for all hospitals. For example, access block post-FHR reduced by up to 13.2% per quarter (Rate Ratio 0.868; 95%CI 0.814, 0.925) for Hospital C, on top of its pre-FHR increasing rate of 3.5% per quarter (Rate Ratio 1.035, CI 1.008, 1.062). Hospitals A and B also demonstrated an immediate reduction in access block in the first quarter of the FHR introduction at a rate of 28.3% and 31.4% respectively.

### Secondary measures

#### Time to being seen by ED clinician

As shown in Table 2, median time to being seen by an ED clinician had an increasing trend pre-FHR and decreasing post-FHR for Hospitals A and D. Hospital A also observed an immediate reduction in this measure by nearly 15 minutes when the FHR was first introduced. An opposite pattern of change was observed for Hospital C, where median time to initial assessment was on a declining trend pre-FHR, and on an increasing trend post-FHR. The FHR did not have any significant effect on this outcome measure for Hospital B (where the outcome remained unchanged throughout the entire study period) and Hospital E (where the measure was on an increasing trend pre-FHR).

#### ED efficiency

ED efficiency, a categorical version of EDLOS, improved significantly post-FHR for the four hospitals A through D. The largest magnitude of improvement was observed for Hospital D, with an increase in ED efficiency by 2.1% per quarter (see Table 2). For Hospital E, pre-FHR ED efficiency was already on an improving trend at a rate of 0.2% per quarter, and despite an immediate decrease at the beginning of FHR, this trend remained relatively unchanged throughout the post-FHR.

#### Disposition status: Admissions, short-stay admissions and Did-Not-Wait

Trends in admission increased post-FHR for Hospitals C and E, who had decreasing trends pre-FHR (see Table 2). The magnitude of change pre-FHR was the same for the two hospitals, showing a reduction of 0.2% in admission rate per quarter. Post-FHR admission rate at Hospital E increased substantially, by 0.8% per quarter, in addition to the pre-existing downward trend. For Hospitals B and D, there was a statistically significant change in admissions immediately following the FHR, notably an increase of nearly 7% admission rate for Hospital D in the first quarter of post-FHR. Admission rate was stable across different time periods for Hospital A.

The increase in admission rate at Hospital D described above was also reflected in its short-stay admission rate increase immediately post-FHR (Table 2). There was no other significant effect of the FHR on short-stay admissions for any of the participating hospitals. Nevertheless, there was an increasing trend in short-stay admissions pre-FHR at Hospitals C and D.

Did-Not-Wait (DNW) rate was already on a declining trend pre-FHR for Hospitals A and E (Table 2). The FHR brought about an immediate further reduction in DNW rate at Hospital A by 1.2% in the first quarter. However, introduction of the FHR was associated with an increasing trend in DNW rate for Hospital E, by 0.2% per quarter, against its pre-existing declining trend. Hospital D’s DNW rate post-FHR decreased by 0.1% per quarter. No other statistically significant effect was observed.

## Discussion

### Principal findings

Collectively speaking, the FHR produced improvements in several areas of ED functioning. These include: shortening ED length of stay and enhancing ED efficiency for most hospitals; alleviating access block and ED overcrowding dramatically for all hospitals; reducing time to be seen by ED clinicians; and reducing rates of ED re-attendance and DNW in some hospitals.

The most consistent and substantial benefits of the FHR program were observed in the ‘flow’ measures, especially access block. This was expected, given that the primary objective of the program was to improve ED and hospital efficiency and reduce congestion and patients waiting. Also notable was the stable, or even downward, trend in ED re-attendance within 7 days. This provides some indication that improvements in flow were not made at the expense of patient care, a concern noted from the UK FHR experience [28]. Nevertheless, we also acknowledge that other, more pertinent measures of unintended adverse patient outcomes (e.g., prolonged total hospital stay, mortality) were not explored in this paper.

Conversely, it is also interesting to note that, while the FHR could be seen as a time-based target, its effect on the two time-based outcome measures, namely ED length of stay and time to be seen by ED clinician, were less uniform than that on the ‘flow’ measures. Broken down by acuity (ATS) categories, the data showed that: ED length of stay for lower acuity (ATS 4 and 5) attendances were already low, and hence remained unchanged with the FHR. The majority of improvement in ED length of stay was seen in the higher acuity levels (ATS 1 through 3) attendances (S1 Fig). By contrast, time to be seen by ED clinician was already short for the highest acuity (ATS 1 and 2) attendances, and hence exhibited no apparent improvement in association with the FHR (S2 Fig). This is to be expected as clinical resources are directed to higher priority patients. The same preliminary analysis (S2 Fig) also suggested that time to initial ED assessment for lower acuity (ATS 4 and 5) attendances may have improved with the FHR at the expense of moderate acuity (ATS 3) attendances for some hospitals. These observations warrant a specific focus of inquiry.

Admission rates increased for most hospitals in post-FHR and in one hospital this trend was accompanied by an increase in short-stay admissions. There had been some clinical concern, documented in the international literature (and commented locally) that efforts to meet the FHR target could lead to a somewhat ‘strategic’ increase in admissions, particularly admissions for low-severity ED attendances, in order to ‘stop the clock’[29]. This would result in a ‘dilution’ effect in admission practice. However, preliminary investigation of our admission data (S3 Fig) based on the ATS categories, provides no basis for this concern. In fact, there seemed to be an increased admission of higher acuity (ATS categories 1–3) cases.

Rate of ED attendance on the whole was already increasing over time, independently of the FHR, except for a significant upward trend observed post-FHR for Hospital A, whilst it’s pre-FHR trend was stable, unlike most other participating hospitals. It is interesting and worth noting that the attendance rate did not increase uniformly as a result of the FHR and its publicity. Similarly, there was no evidence for the ‘dilution’ effect [29] in the acuity of ED attendances due to the FHR (S4 Fig).

### Strengths and weaknesses of the study

Despite over a decade since the UK FHR was introduced, and several similar interventions being introduced worldwide to alleviate the overcrowding and related issues faced by EDs, little systematic research has been published in the peer-reviewed literature [30]. Also previous studies were not focused on a comprehensive range of ED performance indicators like ours, and were limited to fewer selected outcomes, such as ED length of stay [31, 32], DNW rate [33], overcrowding and access block [34] and mortality [21, 31, 32, 34, 35]. Further buttressing this strength, our study presents the longest longitudinal series of data in the field (12 years), with data from five sites with different profiles. Our study applied the ITS statistical technique to more accurately evaluate changes in the outcome measures that are attributable to the FHR.

Another strength of our study is the large data coverage, which has allowed us to reliably measure ED occupancy over time for each participating hospital, hence providing an accurate estimate of the extent of ED overcrowding throughout the long study period. ED overcrowding represents a key aspect of the ED and hospital environment, which should be of significant value in subsequent related studies in assessing specific patient outcomes of interest, such as: “Does presenting to an overcrowded ED and hospital environment put the patient at significantly higher risk of mortality?” Some previous studies applied a perhaps less informative proxy measure for ED overcrowding (e.g., via access block [34, 36]); while others attempted to measure the construct using a more original method.[4, 5] Nonetheless, to our knowledge, none of these estimates has the combined strength of accuracy and durability as demonstrated in our study.

These strengths are juxtaposed by two shortcomings. First, while reporting on an extensive range of ten outcome measures related to ED and hospital functioning, we needed to restrict our scope of analysis to time trends and intervention effects only (and where particularly relevant, triage categories), in order to fit into the scope of one paper. As such, examination of factors considered important such as patient age, comorbidity, mode of transport, socio-economic disadvantage, as well as system and environmental factors such as ED shift, ED environment (e.g., extent of overcrowding) and seasonal factors, and their interplay with the intervention effect, have not been included here. Second, with WA being the first Australian state to implement the FHR–hence acquiring a lengthy series of longitudinal data, it is not currently possible to include a similarly long series of data from hospitals in other Australian jurisdictions. Consequently, with the five sites under investigation, the level of homogeneity may not have been ideal, which is why we presented the results for the five sites separately. Hence, the generalisability of our findings may be limited [21].

Another limitation in this study is its primary focus on the intervention’s impact on ED performance indicators, and this focus is not extended to ‘downstream’ effects on specific inpatients’ outcomes, except for the study’s examination of admission and access block measures, which concern inpatients. On this point, we also note some recent literature examining such issues. For example, Perera and colleagues [37, 38] reported positive and negative outcomes such as reduction in ED waiting times and increased use of short stay units as well as prolonged inpatient stays and an increased rate in inter-unit transfers for certain subgroups of inpatients (e.g., those utilizing acute surgical services) after the FHR intervention. Our findings are consistent with these studies; however, we have not reported on specific conditions as it was beyond the scope of the study.

### Explanations and implications of the study’s findings

Despite between-hospital variations in certain outcomes as noted earlier, the FHR experience in WA has overall been positive for the observed measures. This reflects the potential value of investing resource and effort into the hospital system resulting in improved functionality, which improves the patient experience (at least in the ED environment). Although mortality outcome has not been assessed in this report, we have addressed some important clinical questions, including whether the FHR (i) brought about unintended adverse, or suboptimal consequences such as increased ED re-attendance; (ii) was associated with a dilution effect in acuity of ED presentations; (iii) ‘bed-shuffling’ of patients just to ‘stop the clock’ and meet the time-based target by increasing admissions, and particularly short-stay admissions. As summarised in Results and discussed in the Principal Findings section earlier, we found no clear evidence from our study to serve as a basis for any of these concerns, which is positive.

Our results also suggest that the FHR in WA was not regarded simply as a time-based target by the participating WA hospitals. Rather, it was a major driver or catalyst for system and practice changes on many levels. For example, for some hospitals, despite demonstrating modest changes in their median EDLOS, their other ED experiences such as access block and ED overcrowding, still benefited from adopting the intervention. Changes and improvements, to some extent, were different for different participating hospitals, depending on their inherent structure and setup (e.g. public or private funding, tertiary or non-tertiary, treating adults or children or mixed) and context (e.g. city centre or suburban, patient demography and socio-economic disadvantage), to name a few. Accordingly, these factors need to be taken into account when attempting to translate this study’s findings into meaningful changes to policies and practices for other hospitals.

### Future research

We are exploring the exact mechanisms of the introduction and effects of the FHR on the ED and hospital functioning such as mortality, comorbidities, and other variables described above, as well as the national impact. This is important mainly for two reasons: (a) to assess further the question of any FHR unintended adverse consequences, and (b) to compare with preliminary national and international findings [1, 21, 30, 39]. To meaningfully translate our research findings to clinical and administrative changes, we consider that simulation modelling will be a useful tool.

## Supporting information

S1 File Additional information and illustration of ITS technique in SAS. (DOCX)

S1 Fig Summary of raw data for median ED length of stay (measured in hours), with breakdown by Australasian Triage Score (ATS) categories: Highest acuity (ATS 1&2) attendances; Moderate Acuity (ATS 3) attendances; and lower acuity (ATS 4&5) attendances. (TIF)

S2 Fig Summary of raw data for median time to being seen by ED clinician (measured in minutes), with breakdown by Australasian Triage Score (ATS) categories: Highest acuity (ATS 1&2) attendances; Moderate Acuity (ATS 3) attendances; and lower acuity ATS 4&5 attendances. (TIF)

S3 Fig Summary of raw data for admission rate, with breakdown by Australasian Triage Score (ATS) categories: Highest acuity (ATS 1&2) attendances; Moderate Acuity (ATS 3) attendances; and lower acuity (ATS 4&5) attendances. (TIF)

S4 Fig Summary of raw data for ED attendance rate, with breakdown by Australasian Triage Score (ATS) categories: Highest acuity (ATS 1&2) attendances; Moderate Acuity (ATS 3) attendances; and lower acuity (ATS 4&5) attendances. (TIF)

S1 Acknowledgments (PDF)
