# Supplementary material for: Impact of the Four-Hour Rule in Western Australian hospitals: Trend analysis of a large record linkage study 2002-2013
Source: PLoS One. 2018 Mar 14;13(3):e0193902. doi: 10.1371/journal.pone.0193902 (PMC5851625; doi:10.1371/journal.pone.0193902)
Supplement: S1 File — (DOCX) [file pone.0193902.s001.docx]

**Supplementary File**

**Interrupted times series (ITS) analysis: a brief description and example**

An interrupted time series (ITS) analysis sometimes known as quasi-experimental time series analysis is a quantitative statistical method of a single time series of data known to be interrupted by an intervention at a known point in time [[1-3](#_ENREF_1)]. In an ITS approach, a series of observations on the same outcome before and after the introduction of an intervention are used to test immediate and gradual effects of the intervention.[[4](#_ENREF_4), [5](#_ENREF_5)] A major strength of ITS method is its ability to distinguish the effect of the intervention from secular change, that is, change that would have happened even in the absence of the intervention. The estimation of the intervention effect is done by comparing the trend in the outcome after the intervention to the existing trend in the pre-intervention period, and is achieved through modifications to the standard regression analysis which is known as segmented regression analysis [[1-3](#_ENREF_1)]. In the segmented regression, the time period is divided into ‘pre- and post-intervention’ segments, and separate intercepts and slopes are estimated in each segment. Statistical tests of changes in intercepts and slopes pre to post-intervention are carried out. The basic segmented regression analysis can be written as [[1-3](#_ENREF_1)]:

*Y_t_ = β_0_ + β_1_Time_t_ + β_2_ (Intervention_t_ ) + β_3_ (Time after intervention_t_ ) + e_t_*

*Y_t_* is the outcome at time ‘*t*’;

*‘Time’* indicates the number of quarters from the start of the series;

*‘Intervention’* is a dummy variable taking the values ‘0’ for pre-intervention and ‘1’ for post-intervention segment;

*‘Time after intervention’* is taking values ‘0’ in the pre-intervention and counts the quarters in the post-intervention segment at time ‘*t’;*

*β_0_* estimates the base level of the outcome at the beginning of the series (at time ‘t’ = 0);

*β_1_* estimates the change in outcome per quarter in the pre-intervention segment;

*β_2_* estimates the change in level in the post-intervention segment;

*β_3_* estimates the change in trend in the post-intervention segment; and

*e_t_* estimates the error.

For our data ITS analysis segmented regression can be written as:

*Y_t_ = β_0_ + β_1_*  (QuartNo)*_t_ + β_2_*  (FHR)*_t_ + β_3_ (Quart_Post)_t_ + e_t_*

Where ***Y_t_***  represents the ‘Median EDLOS’ as the outcome variable (Hospital D as an example);

QuartNo (for trend pre-intervention);

FHR (for change in level, immediately after intervention); and

Quart_Post (for change in trend post-intervention).

For ITS analysis we used SAS Statistical software and SAS outputs presented in Table A to C below.

**Sample SAS code and relevant output for the Interrupted Time Series (ITS) model for Hospital D’s median EDLOS**

**SAS code**

**proc** **autoreg** data=normEDLOS outest=paraEDLOS covout;

where hospital = D;

model MedEDLOS = QuartNo FHR Quart_Post / method = ml nlag=**5** backstep dwprob loglikl covb;

output out=predEDLOSnorm p=predict r=resid;

**run**;

PROC AUTOREG invokes the procedure for ITS modeling. “data” specifies the dataset **normEDLOS** for analysis.

“where” restricts the analysis to Hospital D only.

“model” statement specifies: MedEDLOS as the outcome variable; and QuartNo (for trend pre-intervention), FHR (for change in level, immediately after intervention), and Quart_Post (for change in trend post-intervention) as the three explanatory variables, required for a standard ITS model. See also notations for Table A below.

All the other options are optional/modifiable, depending on the actual data being modeled and the researcher’s preference. For example, “nlag” specifies 5 data points, to make up 4 intervals, or quarters in our dataset, to account for seasonality.

**SAS output**

Table A: Estimates of coefficients from ITS model

Intercept represents the starting point of the data series, which is not of interest to this study (hence not reported in Table 2 in the main manuscript).

On the other hand, estimates for the next three terms: QuartNo, FHR, and Quart_Post are reported in the relevant section in Table 2 in the main manuscript. Their standard errors are used to compute the associated 95% Confidence Intervals, also reported in Table 2.

QuartNo represents trend (or slope) in the time series before intervention. Here, the model suggests that, pre-intervention, median EDLOS for Hospital D increased at a rate of approximately 0.015 hours (or 0.9 minutes) per quarter. However, this trend estimate was not statistically significant (i.e., not different from zero).

FHR represents the segmentation between before- and after-intervention, namely, the change in level. Here, the immediate change in level in the first quarter just after the intervention was estimated to be an increase of 0.159 hours (or 9.5 minutes); again, this was not statistically significant.

Quart_Post represents the change in trend (or slope) after intervention. Here, the model suggests a significant decrease in trend in median EDLOS post-intervention, by 0.139 hours (or 8.3 minutes) per quarter. This means that the estimated trend for post-intervention was estimated to be 0.015 (trend pre) – 0.139 (trend post) = -0.124 (i.e., a reduction of around 7 minutes in median EDLOS per quarter).

AR1, AR4, and AR5 represent the auto-correlations at lags 1, 4, and 5, respectively. These terms, when significant, indicate seasonality in the data series. They, however, are not considered part of the ‘structural’ components of the ITS model, as the preceding four terms are.

**Goodness of Fit measures – Numerical**

Table B: Some indexes of model fit based on maximum likelihood method (chosen)

Table C: Some indexes of model fit, based on ordinary least square method (not chosen)

Model fit indexes from Table B (chosen) are superior to those in Table C, with the former showing lower Root Mean Square Error (RMSE), lower corrected Akaike Information Criterion (AICC), and higher Total R-Square.

**Goodness of Fit measures – Graphical**


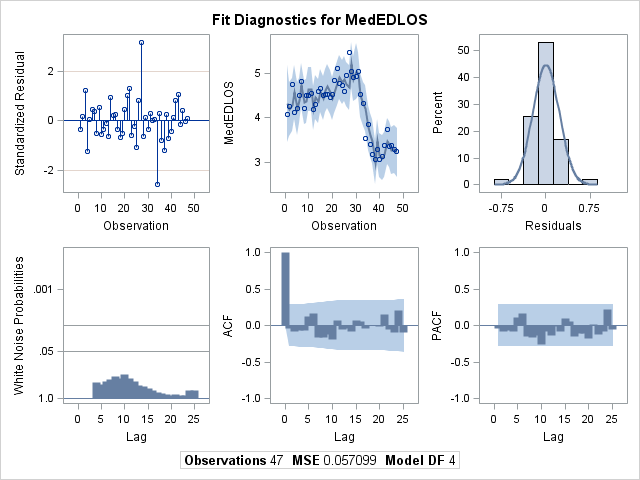


The first graph shows the standardized residuals from the ITS model lie within the acceptable range of [-2, +2], except for two quarters only.

The second graph shows a reasonable model fit (the gray lines) along the actual data (the blue circles). The shaded area indicates the 95% confidence band for the model fit.

The third graph shows the model’s residuals almost conforming to a random normal distribution, with mean 0.

The fourth graph indicates high probabilities (>0.05) of the residuals being white noise.

The fifth graph is for auto-correlation functions. It shows that autocorrelations between data points at different lags fall into the acceptable/shaded area, except for the data at lag 0 – that is, the data at any time point correlate 100% with themselves, which is obvious.

The last graph is for partial auto-correlation functions. It shows that all the correlations between any two data points, taking into account all the lags in between them, fall into the acceptable range.

All the above combined indicate a satisfactory model fit for Hospital D’s median EDLOS measure.

**References**

1. Wagner, A.K., et al., *Segmented regression analysis of interrupted time series studies in medication use research.* Journal of Clinical Pharmacy & Therapeutics, 2002. **27**(4): p. 299-309.

2. Penfold, R.B. and F. Zhang, *Use of interrupted time series analysis in evaluating health care quality improvements.* Academic pediatrics, 2013. **13**(6 Suppl): p. S38-44.

3. Gebski, V., et al., *Modelling interrupted time series to evaluate prevention and control of infection in healthcare.* Epidemiol Infect, 2012. **140**(12): p. 2131-41.

4. Aickin, M. and H. Gensler, *Adjusting for multiple testing when reporting research results: the Bonferroni vs Holm methods.* American Journal of Public Health, 1996. **86**(5): p. 726-728.

5. Perneger, T.V., *What's wrong with Bonferroni adjustments.* BMJ, 1998. **316**(7139): p. 1236-8.
