# Supplementary figures and images for: Impact of the Four-Hour Rule in Western Australian hospitals: Trend analysis of a large record linkage study 2002-2013
Source: PLoS One. 2018 Mar 14;13(3):e0193902. doi: 10.1371/journal.pone.0193902 (PMC5851625; doi:10.1371/journal.pone.0193902)

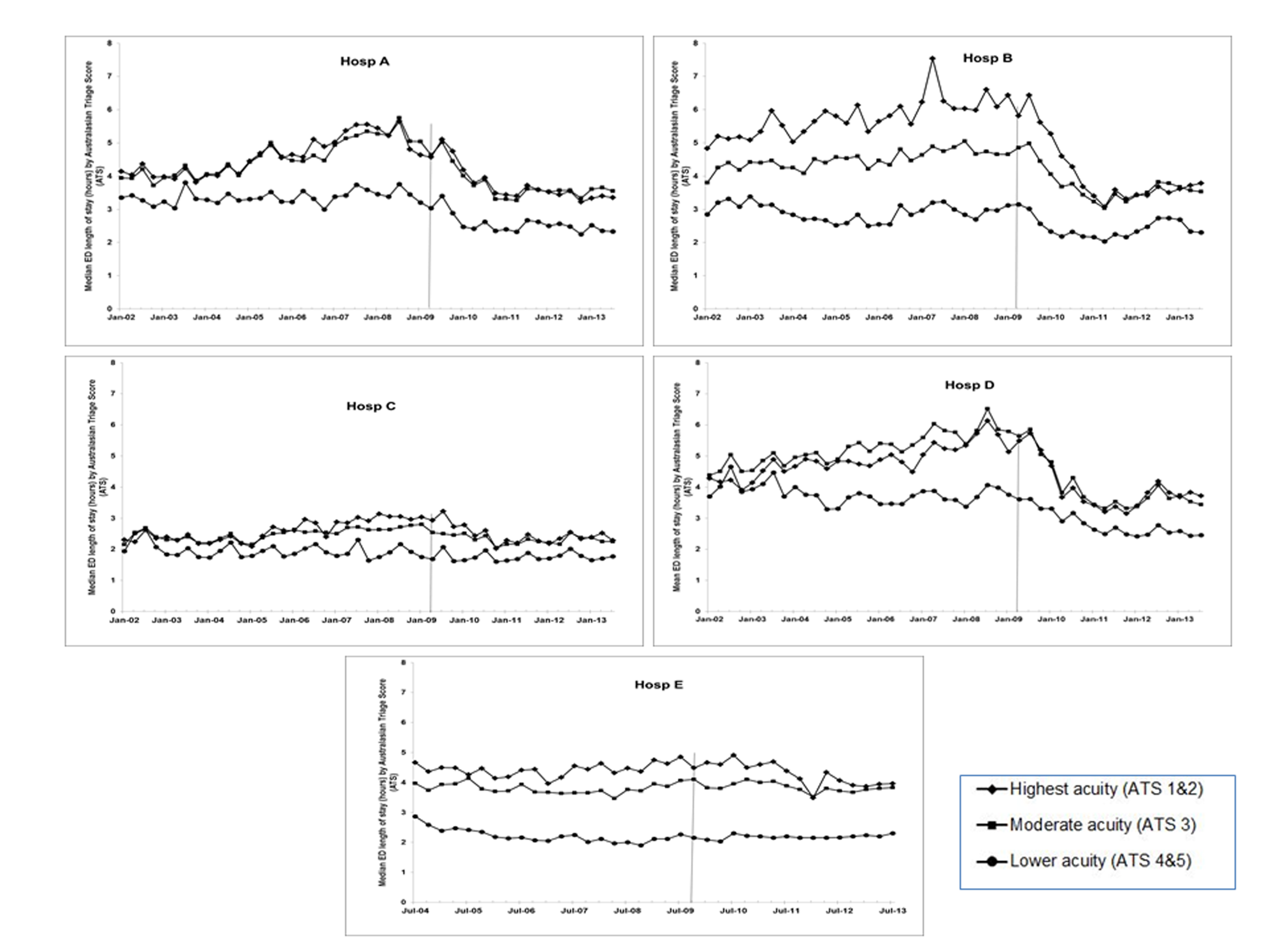

Supplement: S1 Fig — (TIF) [file pone.0193902.s002.tif]

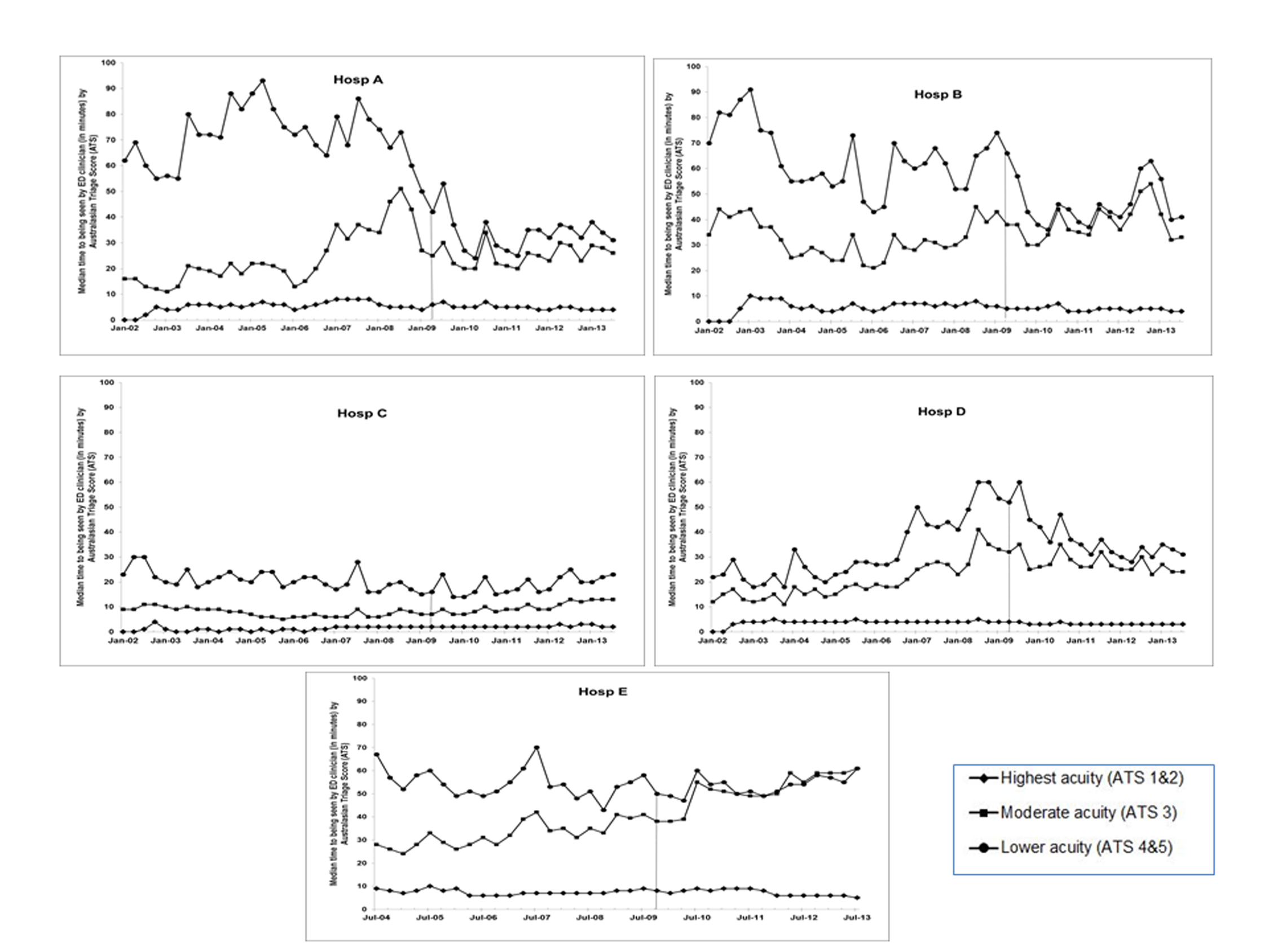

Supplement: S2 Fig — (TIF) [file pone.0193902.s003.tif]

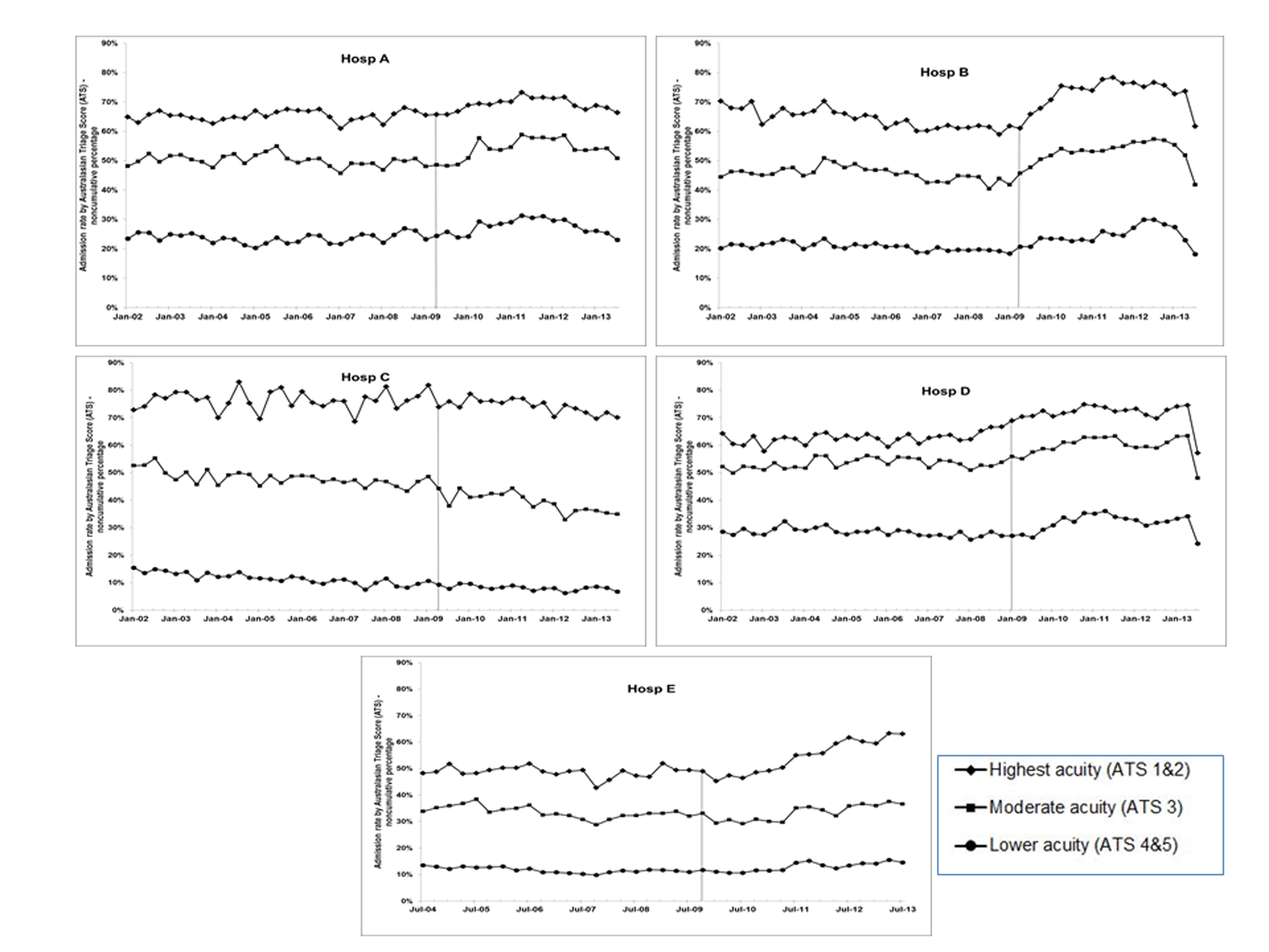

Supplement: S3 Fig — (TIF) [file pone.0193902.s004.tif]

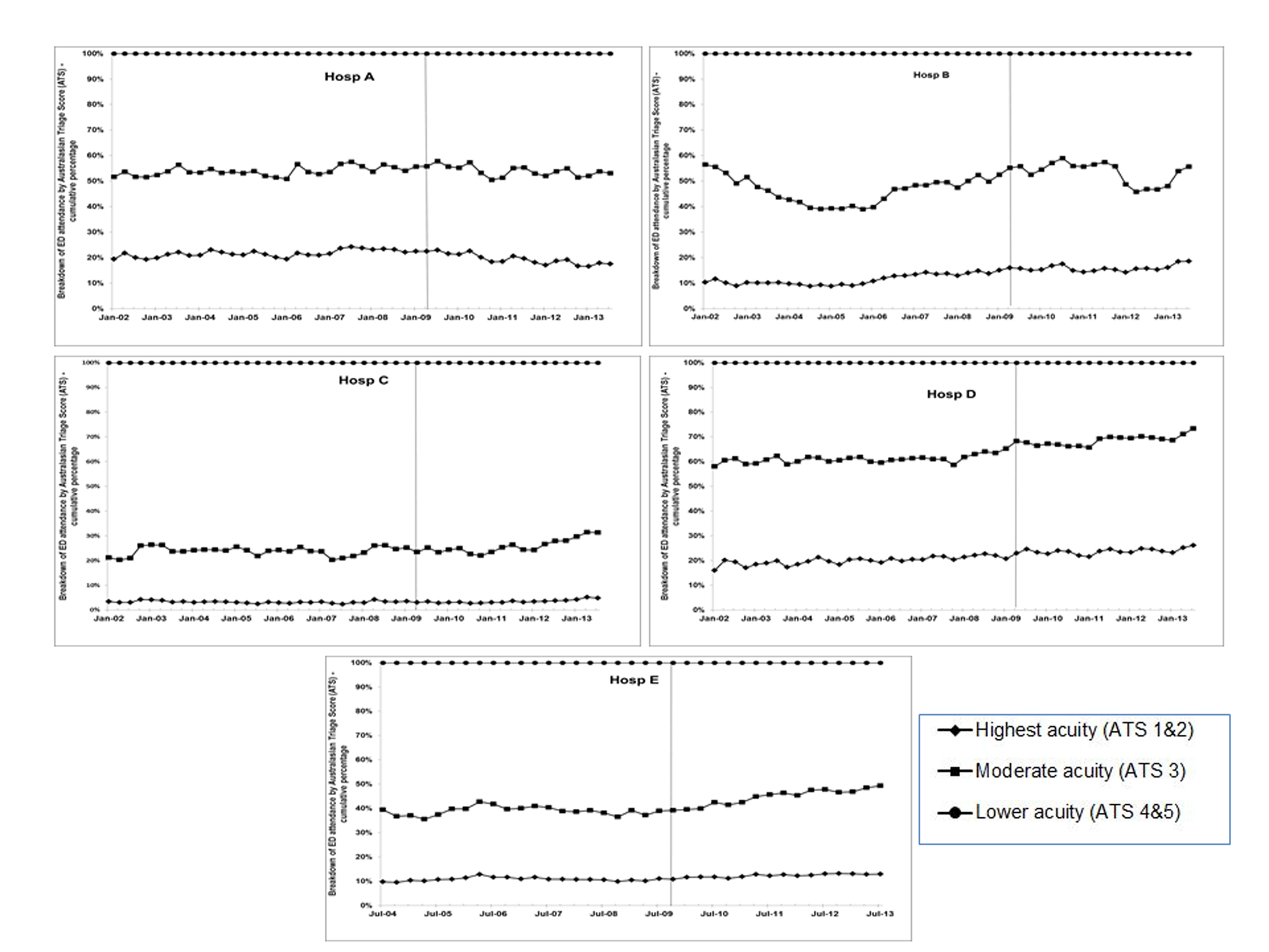

Supplement: S4 Fig — (TIF) [file pone.0193902.s005.tif]
